# The Zebrafish Breathes New Life into the Study of Tuberculosis

**DOI:** 10.3389/fimmu.2016.00196

**Published:** 2016-05-19

**Authors:** Henna Myllymäki, Carina A. Bäuerlein, Mika Rämet

**Affiliations:** ^1^BioMediTech, University of Tampere, Tampere, Finland; ^2^Department of Pediatrics, Tampere University Hospital, Tampere, Finland; ^3^Department of Children and Adolescents, Oulu University Hospital, Oulu, Finland; ^4^PEDEGO Research Unit, Medical Research Center Oulu, University of Oulu, Oulu, Finland

**Keywords:** tuberculosis, zebrafish model system, vaccination, *Mycobacterium marinum*, *Mycobacterium tuberculosis*, *Mycobacterium* infections, granuloma, latency

## Abstract

Tuberculosis (TB) is a global health emergency. Up to one-third of the world’s population is infected with *Mycobacterium tuberculosis*, and the pathogen continues to kill 1.5 million people annually. Currently, the means for preventing, diagnosing, and treating TB are unsatisfactory. One of the main reasons for the poor progress in TB research has been a lack of good animal models to study the latency, dormancy, and reactivation of the disease. Although sophisticated *in vitro* and *in silico* methods suitable for TB research are constantly being developed, they cannot reproduce the complete vertebrate immune system and its interplay with pathogens and vaccines. However, the zebrafish has recently emerged as a useful alternative to more traditional models, such as mice, rabbits, guinea pigs, and non-human primates, for studying the complex pathophysiology of a mycobacterial infection. The model is based on the similarity between *Mycobacterium marinum* – a natural fish pathogen – and *M. tuberculosis*. In both zebrafish larvae and adult fish, an infection with *M. marinum* leads to the formation of macrophage aggregates and granulomas, which resemble the *M. tuberculosis* infections in humans. In this review, we will summarize the current status of the zebrafish model in TB research and highlight the advantages of using zebrafish to dissect mycobacterial virulence strategies as well as the host immune responses elicited against them. In addition, we will discuss the possibilities of using the adult zebrafish model for studying latency, dormancy, and reactivation in a mycobacterial infection.

## Introduction

Tuberculosis (TB) is still the world’s second deadliest infectious disease killing 1.5 million people and with an estimated 9.6 million new cases reported to the WHO in 2015 (1). An estimated one-third of the world’s population has been exposed to TB. 5–10% of these latent carriers will eventually develop the active disease (1).

**Figure 1:**
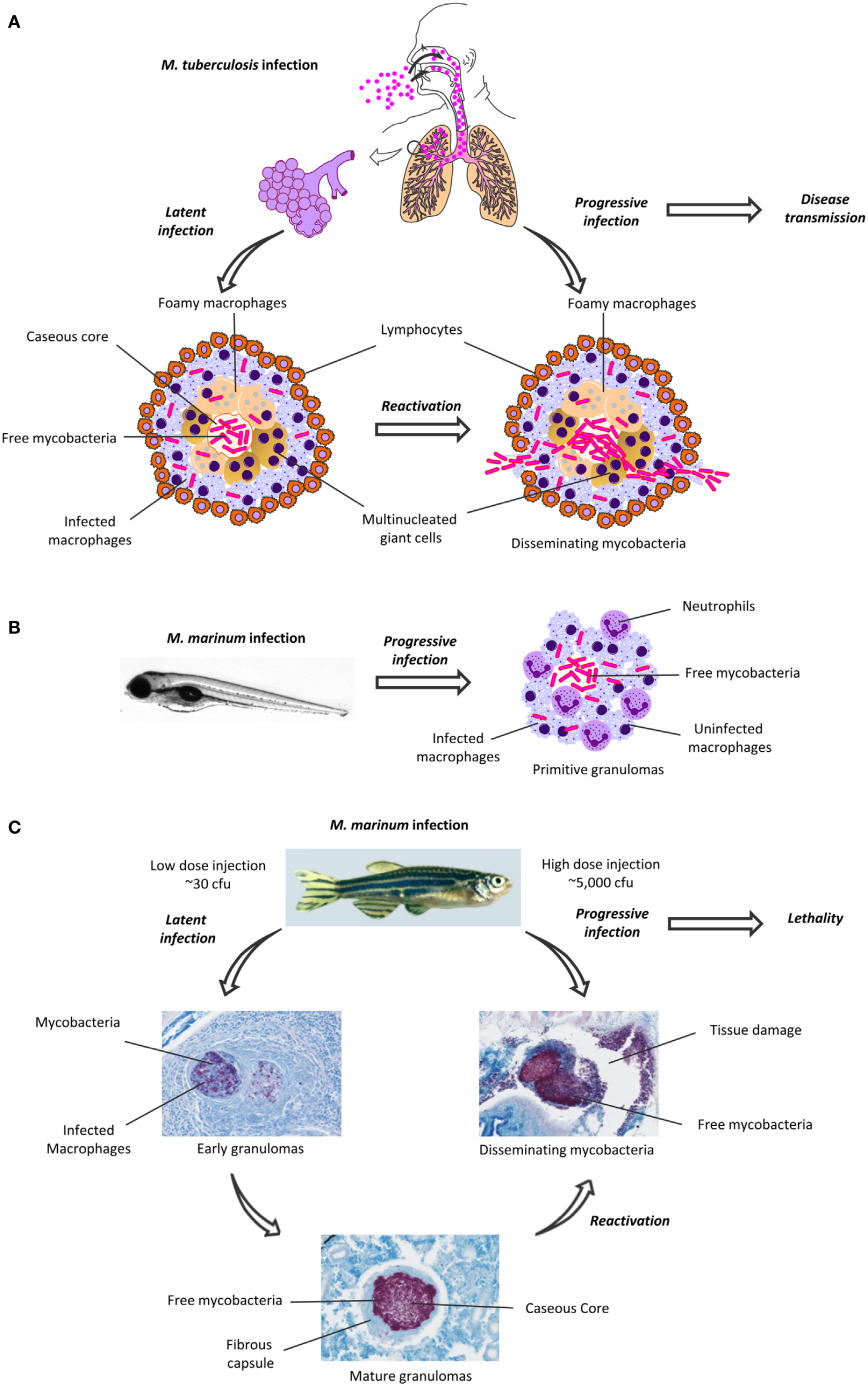
**Granuloma formation in human *Mycobacterium tuberculosis* infection and in zebrafish *M. marinum* infection**. **(A)** *M. tuberculosis* spreads as an aerosol, and first infects alveolar macrophages. In most individuals, the infection is maintained in a latent, subclinical state, which is characterized by the formation of granulomas (left). The mature granulomas have a caseous, necrotic core, surrounded by infected macrophages and lymphocytes. Upon reactivation, the granulomas are disrupted, causing cavities in the lungs. The mycobacteria escape from the granulomas and are disseminated in cough droplets, which facilitates the transmission of the disease. **(B)** Upon infection with *M. marinum*, the granulomas in the zebrafish embryo develop within a few days and mainly consist of infected and uninfected macrophages and recruited neutrophils. **(C)** Depending on the bacterial dose, the adult zebrafish *M. marinum* infection can lead to a latent or an active, progressive disease. A latent infection is characterized by the formation of granulomas in various organs. As the early granulomas mature, their inner parts become caseous and surrounded by a fibrous wall. The zebrafish with a latent infection remain asymptomatic. A (re)activated, progressive mycobacterial infection is characterized by the disruption of the granulomatous structures, rapid replication and dissemination of mycobacteria and profound tissue damage. Eventually, a progressive mycobacterial disease will lead to death in most fish. The zebrafish granulomas were visualized with Ziehl–Neelsen staining, mycobacteria are seen as purple rods. cfu, colony-forming unit.

The causative agent of TB, *Mycobacterium tuberculosis*, spreads through the air (Figure 1A). Alveolar macrophages phagocytose the inhaled mycobacteria and transport them into the lung tissues (2). A cascade of pro- and anti-inflammatory signaling leads to the recruitment and accumulation of additional macrophages and other leukocytes in the pulmonary tissues. Eventually, the formation of granulomas, the hallmark of pathological TB, is initiated. The granuloma is a heterogeneous, but well-organized, and dynamic accumulation of immune cells, including blood-derived infected and uninfected macrophages, foamy macrophages, and epithelioid cells (3). The inner cell mass is usually surrounded by a ring of leukocytes and fibroblasts (4) (Figure 1A; Table 1). The localization and control of the bacteria and the restriction of the immune response to a defined area are generally regarded as the main functions of granulomas (5).

**Table 1:** **Characteristics of a mycobacterial infection in humans, adult zebrafish, and zebrafish embryos**.

	Human	Adult zebrafish (>3 months)	Zebrafish embryo (<6 days)	Reference
**Immune system**	Innate and adaptive	Innate and adaptive	Innate only	(6–8)
Mycobacterial pathogen	*Mycobacterium tuberculosis* and atypical mycobacteria including *Mycobacterium marinum*	*Mycobacterium marinum*	*Mycobacterium marinum*	(9–11)
Natural susceptibility	Yes	Yes	Yes	(9, 10, 12)
Infection route and infectious dose		**Natural infection**		
	Airways ID_50_ <10 bacilli	Digestive tract	?	(9, 10, 12)
		**Experimental infection**		
	N/A	Multiple injection techniques, intraperitoneal injection is the most commonly used; <30–10,000 cfu	Multiple injection techniques, caudal injection is the most commonly used; <10–>300 cfu	(8, 13–15)
Infection phases	Acute	Acute	Progressive?	(10, 13–17)
	Latent	Latent		
	Reactivation	Reactivation		
Granuloma types	Early	Early	Primitive?	(4, 5, 13–18)
	Fibrous	Fibrous		
	Necrotic	Necrotic		
Cell types involved in granuloma formation	Macrophages	Macrophages	Macrophages	(4, 5, 13–18)
	Neutrophils	Neutrophils	Neutrophils	
	Dendritic cells	Dendritic cells	Epithelial cells	
	Lymphocytes (T cells, B cells, and NK cells)	Lymphocytes (T cells and B cells)		
	Fibroblasts	Epithelial cells		
	Epithelial cells			

Mycobacteria can persist asymptomatic within the granuloma for decades. However, a dysregulation of the immune system can reactivate the mycobacteria, which leads to disease progression (secondary TB) (2) (Figure 1A) Primary TB mainly occurs in children, who are at the highest risk for TB meningitis and a disseminated form of the disease (19).

Efforts to eradicate TB are obstructed by the lack of unambiguous diagnostic tools, the lengthy antibiotic treatments required for curation, the growing problem of multi-drug-resistant bacteria, and the poor protection provided by the Bacillus Calmette–Guérin (BCG), the only vaccine available (1). As a live vaccine, BCG imposes a risk of a disseminated infection in immunocompromised patients (1, 20, 21). Thus, there is a need to develop new effective drugs and vaccines against TB. For this purpose, relevant animal models are essential. The most commonly used animal models in TB research are mice, guinea pigs, and non-human primates (NHP), all of which have their limitations related to either space, costs, ethical aspects, or their ability to replicate the human disease pathology (22, 23). Recently, the zebrafish–*M. marinum* model has gained popularity as a natural pathogen–host system that closely recapitulates the pathology of human TB (Table 1) (13, 23). The infection model and its applications are discussed in more detail below.

## The Zebrafish–*Mycobacterium marinum* Infection Model

*Mycobacterium marinum*, the causative agent of fish mycobacteriosis, is a close relative of *M. tuberculosis* (24). *M. marinum* spreads *via* water, and it also occasionally infects humans, but the infection is usually limited to the skin (fish tank granuloma) (11). Thus, *M. marinum* is safer to work with and has a shorter replication time than *M. tuberculosis* (9, 23). Similar to human TB, fish mycobacteriosis displays an acute and chronic form and the subsequently formed granulomas also resemble the lesions caused by *M. tuberculosis* [Figure 1 (13–15)]. Both bacteria are able to survive and replicate within macrophages (23, 25). In a laboratory setting, the zebrafish is an advantageous choice as a host organism for *M. marinum* for several reasons: multiple infection techniques can be used for both zebrafish embryos and adults (26); for a review, see Ref. (8, 27–29) (Figures 1B,C). The transparency of the embryos allows the use of sophisticated *in vivo* real-time imaging techniques, including multiple leukocyte and macrophage fluorescent reporter lines (30–32), and several techniques for genetic manipulation (6, 33–35). Moreover, zebrafish are small in size, and produce numerous offspring, making them also suitable for large-scale screening studies, including drug screens (6). Despite the anatomical differences between fish and mammals, the zebrafish is a vertebrate model with an innate and adaptive immunity consisting of the same primary components as present in humans (6, 36, 37). As zebrafish lack lymph nodes, immune cells mainly develop and perform their functions in the spleen, the kidney, and the thymus (6, 38, 39). Zebrafish embryos rely solely on innate immunity. In the embryos, functional macrophages and neutrophils emerge 1 and 2 days post-fertilization (dpf), respectively, while lymphocytes start developing after 4 dpf and the adaptive immunity becomes fully functional at 4 weeks post-fertilization (wpf) (7). This facilitates studying the function of the innate and adaptive immune system, as well as different cell types, separately (8, 33).

## Host–*Mycobacterium* Interactions – Insights into Early Infection Events from Zebrafish Larvae

Zebrafish larvae have been especially useful in elucidating the role of macrophages and the strategies the phagocytosed mycobacteria use to suppress phagosomal maturation, apoptosis, and the antibacterial innate immune response (8, 25, 40). Scavenger receptors of different classes have been studied in the context of the phagocytosis of mycobacteria. For example, Marco binds the glycolipid trehalose 6,69-dimycolate on the mycobacterial cell wall and affects the regulation of the subsequent proinflammatory response (41). CD36 also appears to be involved in mycobacterial control, though its role and the regulation of its expression seem quite complicated (42). Following phagocytosis, Toll-like receptor (TLR) signaling *via* MyD88 is needed for resistance against an early mycobacterial infection (43, 44). For example, the activation of the TLR signaling leads to the production of antibacterial molecules by macrophages, such as the perforin Mpeg1.2 (45). In the zebrafish, the *myd88*^−/−^ mutants have been a useful tool in elucidating the role of TLR signaling in mycobacterial resistance (46, 47).

Toll-like receptor signaling is involved in the initiation of the production of reactive oxygen and nitrogen species (ROS and RNS, respectively). In the zebrafish, early stabilization of the transcription factor hypoxia-inducible factor 1α (Hif-1α) in neutrophils limits bacterial growth by inducing iNOS, which in turn leads to increased protein nitrosylation (48). Later in the course of the infection, this response is decreased in the developing granulomas by a mechanism independent of the early secretory antigenic target 6 system 1/region of difference 1 (ESX-1/RD1) virulence locus (49).

As a mean to counteract the mycobacterial evasion strategies, the host can induce autophagy, a process which enables cells to digest their cytoplasmic contents, including microorganisms and membranous structures, in lysosomes (50). This is dependent on TLR signaling and Myd88, which are linked *via* the DNA damage-regulated autophagy modulator DRAM1. DRAM1 is needed for the formation of autophagosomes and for their fusion with lysosomes, while a DRAM1 deficiency leads to defects in maintaining the mycobacteria inside vesicles in macrophages and in the control of mycobacterial growth (51), Again, zebrafish larvae provide feasible tools for observing the events of autophagy *in vivo* and in real-time utilizing both light and electron microscopy, including the GFP-Lc3-transgenic line, for the visualization of autophagosomal structures (52).

## Zebrafish Larvae Model Challenge Old Dogmas in Mycobacterial Resistance

After a successful infection by mycobacteria, granulomas are seeded. The granulomas in zebrafish embryos mainly consist of aggregated macrophages, intra- and extracellular mycobacteria, together with recruited neutrophils, and form within a few days post-infection (18, 53); for a review, see Ref. (54). Despite their rather primitive structure, the embryonic zebrafish granulomas provide a physiological model for studying cellular processes affecting mycobacterial infections, such as the generation of hypoxia and angiogenesis. Injection of mycobacteria into the caudal vein results in the development of non-hypoxic granulomas in the richly vascularized area of the caudal hematopoietic tissue (48). In contrast, granulomas resulting from a trunk infection reside in a sparsely vascularized area and can become hypoxic and induce vascularization (55).

Importantly, studies on early granulomas in zebrafish larvae have challenged some of the old dogmas. In general, granulomas have long been considered a protection mechanism elicited by the host. However, recent evidence from zebrafish embryos suggests that *M. marinum* uses the macrophages and granulomas for its own expansion and dissemination (16, 17). The bacteria can recruit new, uninfected macrophages to the granuloma site using the RD1 locus (16). The arriving macrophages phagocytize infected, dead cells and thus contribute to the spreading of the bacteria. The recruitment of new macrophages is enhanced by the bacteria by ESAT-6-mediated production of matrix metalloproteinase 9 (17). Mycobacteria also need the RD1 locus for escaping from the phagosomes into the cytoplasm of infected macrophages both in human cells and in zebrafish (51, 56). Additionally, mycobacteria use cell surface lipids to mask pathogen-associated molecular patterns, hence selectively infect permissive macrophages and avoid microbicidal ones (57). Thus, granulomas present a combat zone for the host immune system and the bacterial response, rather than purely a protection mechanism of the host to prevent the spread of bacteria (4).

In addition to basic research, the zebrafish larvae provide a feasible tool for early-stage drug development and large-scale screens (33, 58, 59). Two major, and related, issues in improving the TB drug development are the lengthiness of the curative treatments and the increasing emergence of drug-resistant bacteria (1). Discoveries made in zebrafish larvae revealed that intracellular mycobacteria use their efflux pumps to acquire a tolerance against the antibiotics commonly used to treat human TB, which allows the bacteria to persist and replicate in the cytosol. *M. tuberculosis* uses the same mechanism (60). However, this tolerance was reversed by an efflux pump inhibitor, such as verapamil, which can therefore reduce the tolerance to antibiotics and thereby shorten treatment times (61). These results prove that the zebrafish larval model can replicate the function of antitubercular compounds (60). In addition, angiogenesis has been shown to be important for granuloma formation. Therefore, targeting vascularization, for example, by inhibiting vascular endothelial growth factor receptor (VEGFR) signaling could provide a means to target mycobacterial infections and inhibit mycobacterial dissemination, resembling the strategy used in cancer therapies (55).

On the host’s side, cytokines and their respective receptors play an important role in protection against mycobacteria. For example, the signaling axis mediated by chemokine CXC-motive containing receptor 3 (CXCR3) has been implicated in mycobacterial spreading and could thus provide a therapeutic target (62). In addition, in both humans and zebrafish larvae the leukotriene A4 hydrolase (LTA4H) locus controls pro- and anti-inflammatory mediators that in turn control the expression of TNF (63). While TNF is required for the host response against mycobacteria, its excess renders the host more susceptible to an infection, highlighting the importance of a balanced response (64). Thus, the zebrafish can elucidate the pathways controlling the host immune responses, and this information can be further applied to targeting these pathways with new drugs and developing host-directed therapies (8, 63–66). In addition, further investigation into the evasion strategies that mycobacteria use to interfere with the host defense mechanisms can potentially lead to the discovery of novel drug targets to combat mycobacterial diseases (49, 67).

## Mycobacterial Latency vs. Adaptive Immunity – The Adult Zebrafish as a Model for TB

One of the main advantages of the zebrafish–*M. marinum* model may lie in granuloma formation, which has not been easy to reproduce in the traditional model animals (22). In the adult zebrafish, however, the histology of the mature granulomas resembles those seen in human TB with their caseous, necrotic core surrounded by leukocytes and epithelial cells (4, 17). Granulomas are found in various organs such as the pancreas, gonads, spleen, and liver several weeks post-infection (Figure 1C) (13–15).

The latency of TB is another aspect that has been difficult to replicate experimentally (22). As this is also challenging to study in humans, our knowledge of the required immunological mechanisms for the control of a mycobacterial infection at its different stages remains limited (17). However, this could be improved by studying the zebrafish model, as like humans, adult zebrafish develop a latent, non-progressive disease with dormant bacteria residing within well-structured granulomas, and the fish remain asymptomatic (13, 14). Moreover, reactivation of the bacteria can occur spontaneously, or can be induced experimentally by an immune deficiency, such as exposure to γ-irradiation. In either case, reactivation will lead to the active spreading of the bacteria and the development of symptoms similar to an active infection as well as high lethality, much as in human TB (13).

Although the early cytokine response mediated by the innate immunity plays an important role in determining whether a mycobacterial infection leads to an acute or latent disease, the adaptive immunity is also required to control the bacteria. This is seen in the adult *rag*^−/−^ zebrafish that are devoid of lymphocytes. The mutant fish are unable to generate a latent state of the infection and are, therefore, hypersusceptible to *M. marinum* (13, 14, 68). So far, the significance of different lymphocyte subsets has not been extensively studied in the zebrafish mycobacterial infection. Nevertheless, Th1 as well as Th2 cells seem to be involved in the effective control of mycobacterial infections (69, 70). Interestingly, a novel Th2-like subset of cells capable of inhibiting the growth of *M. tuberculosis* has been found in human TB patients. This observation challenges the old idea that only Th1 cells are important for mycobacterial control (71). Genetic differences in the mycobacterial strains also seem to affect their virulence, for example, strains isolated from infected humans more commonly causing an acute disease, and isolates from poikilothermic species causing a chronic infection in the zebrafish (72).

While the zebrafish larvae provide a feasible tool for screening for drugs against TB, the adult zebrafish appears to be a promising model for early vaccine development. The zebrafish can be partially protected against mycobacteriosis by BCG (68, 73) or attenuated *M. marinum* (74), suggesting the use of adult zebrafish as a model for studying the feasibility of conserved mycobacterial antigens as vaccines. For example, the RD1 virulence locus and the ESX-1 secretion system, which are absent from BCG, have been shown to be important for virulence in *M. tuberculosis*. In *M. marinum*, RD1 is also is also required for granuloma formation in both larvae and adult fish (14, 49, 75, 76). Indeed, the antigens in this region do show some potential as targets for vaccines in the zebrafish as well as in other models (68, 73, 77–79). The zebrafish can also provide a feasible model for searching for the most effective antigen combinations and for studying different vaccination strategies. For instance, a DNA-based vaccine consisting of three mycobacterial antigens (Ag85, ESAT-6, and CFP10), which has also been studied in other TB models, confers protection against mycobacteria in zebrafish (68, 80–82). Furthermore, the effect of BCG can be boosted by a DNA vaccine (68, 73, 80–82). Adult zebrafish could therefore be used for developing vaccines for both replacing and boosting BCG, as well as for studying the immunological correlates required for protection.

## Discussion

The lack of an animal model that recapitulates the human disease stages and pathology has in part hampered the development of new drugs, vaccines, and diagnostic tools against TB (22). Even though the mammalian animal models mostly used for TB research, namely, mice, rabbits, and guinea pigs, do develop granulomatous structures, only primates are a natural host for *M. tuberculosis* and show true latency and reactivation (83). However, the use of primates as laboratory animals is difficult in terms of ethical and economic issues as well as space limitations. When drug and vaccine development is considered, a natural host–pathogen pair is likely to be a more reliable model. Moreover, choosing to work with (zebra)fish might have an additional practical advantage: as mycobacterial infections are able to cause epidemics in fish farms, aquariums, and zebrafish facilities (9, 84), vaccinating fish against *M. marinum* is of potential economic and ecological relevance. This has been studied to some extent, for example, in the striped bass with the Ag85A DNA vaccine (85) and in the Japanese flounder with BCG (86). Thus, the results obtained in human biomedical research and veterinary studies could potentially augment each other.

The zebrafish–*M. marinum* infection exhibits essentially the same disease phases as those seen in human TB, including latency and reactivation – either spontaneously or following immunosuppression (13, 14). This might have important implications as the different disease phases probably also represent on the one hand different strategies of bacterial adaptation, and on the other hand, different stages of the host immune response (87). Since these aspects can be replicated in the zebrafish infection, the model should facilitate a more detailed dissection of both the effective (and deleterious) immune responses and the bacterial counter strategies in each stage of the infection. The zebrafish model can also be used to complement the human patient data in the identification of reliable biomarkers for the diagnosis of the different stages of TB (88).

Besides biomarkers, new drugs and vaccines are needed to combat TB. For this, a better knowledge concerning the correlates of a protective immune response is essential (89). The HI virus attacks CD4^+^ T cells, and a co-infection renders the patients highly susceptible to TB. Therefore, it seems that CD4^+^ T cells are important for the host response (90). This does not mean, however, that the study of other cell types should be neglected, as they too can reveal new immunological mechanisms (70, 71). Moreover, deficiencies in the IFN-γ signaling axis lead to hypersusceptibility toward TB, and thus IFN-γ expression has been associated with protection against the disease (90). However, despite inducing high levels of IFN-γ production, a promising vaccine candidate, MVA85A, failed recently to enhance protection in an efficacy trial (91). This suggests that it is unlikely that a single immunological factor could predict the course of a TB infection (90, 92). Thus, there are still gaps in our knowledge of how an effective host defense against TB is elicited, and relevant animal models are needed to fill in the missing information. Furthermore, once the picture of protection mechanisms is more complete, the animal models can aid us in translating this information into the benefit of clinical medicine. We believe the zebrafish will be an important player in fulfilling both of these tasks.

## Author Contributions

All authors contributed to planning and writing the manuscript and designing of the figures.

## Conflict of Interest Statement

The authors declare that the research was conducted in the absence of any commercial or financial relationships that could be construed as a potential conflict of interest.

## References

[1] World Health Organization. Global Tuberculosis Report 2015. (2015). Available from: http://www.who.int/tb/publications/global_report/en/

[2] Frieden TR Sterling TR Munsiff SS Watt CJ Dye C. Tuberculosis. Lancet (2003) 362:887–99. 10.1016/S0140-6736(03)14333-4 13678977

[3] Adams DO The structure of mononuclear phagocytes differentiating in vivo. I. Sequential fine and histologic studies of the effect of Bacillus Calmette-Guerin (BCG). Am J Pathol (1974) 76:17–48. 4601921 PMC1910742

[4] Ramakrishnan L. Revisiting the role of the granuloma in tuberculosis. Nat Rev Immunol (2012) 12:352–66. 10.1038/nri3211 22517424

[5] Adams DO The granulomatous inflammatory response. A review. Am J Pathol (1976) 84(1):164–92. 937513 PMC2032357

[6] Renshaw SA Trede NS. A model 450 million years in the making: zebrafish and vertebrate immunity. Dis Model Mech (2012) 5:38–47. 10.1242/dmm.007138 22228790 PMC3255542

[7] Langenau DM Ferrando AA Traver D Kutok JL Hezel JP Kanki JP In vivo tracking of T cell development, ablation, and engraftment in transgenic zebrafish. Proc Natl Acad Sci U S A (2004) 101:7369–74. 10.1073/pnas.0402248101 15123839 PMC409925

[8] Meijer AH Spaink HP. Host-pathogen interactions made transparent with the zebrafish model. Curr Drug Targets (2011) 12:1000–17. 10.2174/138945011795677809 21366518 PMC3319919

[9] Decostere A Hermans K Haesebrouck F. Piscine mycobacteriosis: a literature review covering the agent and the disease it causes in fish and humans. Vet Microbiol (2004) 99:159–66. 10.1016/j.vetmic.2003.07.011 15066718

[10] O’Garra A Redford PS McNab FW Bloom CI Wilkinson RJ Berry MP. The immune response in tuberculosis. Annu Rev Immunol (2013) 31:475–527. 10.1146/annurev-immunol-032712-095939 23516984

[11] Linell F Norden A *Mycobacterium balnei*, a new acid-fast bacillus occurring in swimming pools and capable of producing skin lesions in humans. Acta Tuberc Scand Suppl (1954) 33:1–84. 13188762

[12] Harriff MJ Bermudez LE Kent ML. Experimental exposure of zebrafish, *Danio rerio* (Hamilton), to *Mycobacterium marinum* and *Mycobacterium peregrinum* reveals the gastrointestinal tract as the primary route of infection: a potential model for environmental mycobacterial infection. J Fish Dis (2007) 30:587–600. 10.1111/j.1365-2761.2007.00839.x 17850575

[13] Parikka M Hammaren MM Harjula SK Halfpenny NJ Oksanen KE Lahtinen MJ *Mycobacterium marinum* causes a latent infection that can be reactivated by gamma irradiation in adult zebrafish. PLoS Pathog (2012) 8:e1002944. 10.1371/journal.ppat.1002944 23028333 PMC3459992

[14] Swaim LE Connolly LE Volkman HE Humbert O Born DE Ramakrishnan L. *Mycobacterium marinum* infection of adult zebrafish causes caseating granulomatous tuberculosis and is moderated by adaptive immunity. Infect Immun (2006) 74:6108–17. 10.1128/IAI.00887-06 17057088 PMC1695491

[15] Prouty MG Correa NE Barker LP Jagadeeswaran P Klose KE Zebrafish-*Mycobacterium marinum* model for mycobacterial pathogenesis. FEMS Microbiol Lett (2003) 225:177–82. 10.1016/S0378-1097(03)00446-4 12951238

[16] Davis JM Ramakrishnan L. The role of the granuloma in expansion and dissemination of early tuberculous infection. Cell (2009) 136:37–49. 10.1016/j.cell.2008.11.014 19135887 PMC3134310

[17] Volkman HE Pozos TC Zheng J Davis JM Rawls JF Ramakrishnan L. Tuberculous granuloma induction via interaction of a bacterial secreted protein with host epithelium. Science (2010) 327:466–9. 10.1126/science.1179663 20007864 PMC3125975

[18] Davis JM Clay H Lewis JL Ghori N Herbomel P Ramakrishnan L. Real-time visualization of mycobacterium-macrophage interactions leading to initiation of granuloma formation in zebrafish embryos. Immunity (2002) 17:693–702. 10.1016/S1074-7613(02)00475-2 12479816

[19] Marais BJ Gie RP Schaaf HS Beyers N Donald PR Starke JR. Childhood pulmonary tuberculosis: old wisdom and new challenges. Am J Respir Crit Care Med (2006) 173:1078–90. 10.1164/rccm.200511-1809SO 16484674

[20] Roy A Eisenhut M Harris RJ Rodrigues LC Sridhar S Habermann S Effect of BCG vaccination against *Mycobacterium tuberculosis* infection in children: systematic review and meta-analysis. BMJ (2014) 349:g4643. 10.1136/bmj.g4643 25097193 PMC4122754

[21] Mak TK Hesseling AC Hussey GD Cotton MF Making BCG vaccination programmes safer in the HIV era. Lancet (2008) 372:786–7. 10.1016/S0140-6736(08)61318-5 18774406

[22] Myllymaki H Niskanen M Oksanen KE Ramet M Animal models in tuberculosis research – where is the beef? Expert Opin Drug Discov (2015) 10:871–83. 10.1517/17460441.2015.1049529 26073097

[23] Tobin DM Ramakrishnan L. Comparative pathogenesis of *Mycobacterium marinum* and *Mycobacterium tuberculosis*. Cell Microbiol (2008) 10:1027–39. 10.1111/j.1462-5822.2008.01133.x 18298637

[24] Stinear TP Seemann T Harrison PF Jenkin GA Davies JK Johnson PD Insights from the complete genome sequence of *Mycobacterium marinum* on the evolution of *Mycobacterium tuberculosis*. Genome Res (2008) 18:729–41. 10.1101/gr.075069.107 18403782 PMC2336800

[25] Barker LP George KM Falkow S Small PL. Differential trafficking of live and dead *Mycobacterium marinum* organisms in macrophages. Infect Immun (1997) 65:1497–504. 9119492 10.1128/iai.65.4.1497-1504.1997 PMC175158

[26] Benard EL van der Sar AM Ellett F Lieschke GJ Spaink HP Meijer AH. Infection of zebrafish embryos with intracellular bacterial pathogens. J Vis Exp (2012) 61:e3781. 10.3791/3781 22453760 PMC3415172

[27] Meijer AH. Protection and pathology in TB: learning from the zebrafish model. Semin Immunopathol (2016) 38(2):261–73. 10.1007/s00281-015-0522-4 26324465 PMC4779130

[28] van Leeuwen LM van der Sar AM Bitter W. Animal models of tuberculosis: zebrafish. Cold Spring Harb Perspect Med (2014) 5:a018580. 10.1101/cshperspect.a018580 25414379 PMC4355259

[29] Cronan MR Tobin DM. Fit for consumption: zebrafish as a model for tuberculosis. Dis Model Mech (2014) 7:777–84. 10.1242/dmm.016089 24973748 PMC4073268

[30] Hall C Flores MV Crosier K Crosier P. Live cell imaging of zebrafish leukocytes. Methods Mol Biol (2009) 546:255–71. 10.1007/978-1-60327-977-2_16 19378109

[31] Renshaw SA Loynes CA Trushell DM Elworthy S Ingham PW Whyte MK. A transgenic zebrafish model of neutrophilic inflammation. Blood (2006) 108:3976–8. 10.1182/blood-2006-05-024075 16926288

[32] Wittamer V Bertrand JY Gutschow PW Traver D. Characterization of the mononuclear phagocyte system in zebrafish. Blood (2011) 117:7126–35. 10.1182/blood-2010-11-321448 21406720

[33] Lohi O Parikka M Rämet M. The zebrafish as a model for paediatric diseases. Acta Paediatr (2013) 102:104–10. 10.1111/j.1651-2227.2012.02835.x 22924984

[34] Hwang WY Fu Y Reyon D Maeder ML Tsai SQ Sander JD Efficient genome editing in zebrafish using a CRISPR-Cas system. Nat Biotechnol (2013) 31:227–9. 10.1038/nbt.2501 23360964 PMC3686313

[35] Varshney GK Sood R Burgess SM. Understanding and editing the zebrafish genome. Adv Genet (2015) 92:1–52. 10.1016/bs.adgen.2015.09.002 26639914

[36] Traver D Herbomel P Patton EE Murphey RD Yoder JA Litman GW The zebrafish as a model organism to study development of the immune system. Adv Immunol (2003) 81:253–330. 14711058

[37] Traver D Paw BH Poss KD Penberthy WT Lin S Zon LI. Transplantation and in vivo imaging of multilineage engraftment in zebrafish bloodless mutants. Nat Immunol (2003) 4:1238–46. 10.1038/ni1007 14608381

[38] Kissa K Murayama E Zapata A Cortes A Perret E Machu C Live imaging of emerging hematopoietic stem cells and early thymus colonization. Blood (2008) 111:1147–56. 10.1182/blood-2007-07-099499 17934068

[39] Lugo-Villarino G Balla KM Stachura DL Banuelos K Werneck MB Traver D. Identification of dendritic antigen-presenting cells in the zebrafish. Proc Natl Acad Sci U S A (2010) 107:15850–5. 10.1073/pnas.1000494107 20733076 PMC2936643

[40] Koul A Herget T Klebl B Ullrich A Interplay between mycobacteria and host signalling pathways. Nat Rev Microbiol (2004) 2:189–202. 10.1038/nrmicro840 15083155

[41] Benard EL Roobol SJ Spaink HP Meijer AH. Phagocytosis of mycobacteria by zebrafish macrophages is dependent on the scavenger receptor Marco, a key control factor of pro-inflammatory signalling. Dev Comp Immunol (2014) 47:223–33. 10.1016/j.dci.2014.07.022 25086293

[42] Fink IR Benard EL Hermsen T Meijer AH Forlenza M Wiegertjes GF. Molecular and functional characterization of the scavenger receptor CD36 in zebrafish and common carp. Mol Immunol (2015) 63:381–93. 10.1016/j.molimm.2014.09.010 25306962

[43] Velez DR Wejse C Stryjewski ME Abbate E Hulme WF Myers JL Variants in Toll-like receptors 2 and 9 influence susceptibility to pulmonary tuberculosis in Caucasians, African-Americans, and West Africans. Hum Genet (2010) 127:65–73. 10.1007/s00439-009-0741-7 19771452 PMC2902366

[44] Kleinnijenhuis J Oosting M Joosten LA Netea MG Van Crevel R. Innate immune recognition of *Mycobacterium tuberculosis*. Clin Dev Immunol (2011) 2011:405310. 10.1155/2011/405310 21603213 PMC3095423

[45] Benard EL Racz PI Rougeot J Nezhinsky AE Verbeek FJ Spaink HP Macrophage-expressed perforins mpeg1 and mpeg1.2 have an anti-bacterial function in zebrafish. J Innate Immun (2015) 7:136–52. 10.1159/000366103 25247677 PMC6738794

[46] van der Vaart M van Soest JJ Spaink HP Meijer AH. Functional analysis of a zebrafish myd88 mutant identifies key transcriptional components of the innate immune system. Dis Model Mech (2013) 6:841–54. 10.1242/dmm.010843 23471913 PMC3634667

[47] van der Sar AM Stockhammer OW van der Laan C Spaink HP Bitter W Meijer AH. MyD88 innate immune function in a zebrafish embryo infection model. Infect Immun (2006) 74:2436–41. 10.1128/IAI.74.4.2436-2441.2006 16552074 PMC1418923

[48] Elks PM Brizee S van der Vaart M Walmsley SR van Eeden FJ Renshaw SA Hypoxia inducible factor signaling modulates susceptibility to mycobacterial infection via a nitric oxide dependent mechanism. PLoS Pathog (2013) 9:e1003789. 10.1371/journal.ppat.1003789 24367256 PMC3868520

[49] Elks PM van der Vaart M van Hensbergen V Schutz E Redd MJ Murayama E Mycobacteria counteract a TLR-mediated nitrosative defense mechanism in a zebrafish infection model. PLoS One (2014) 9:e100928. 10.1371/journal.pone.0100928 24967596 PMC4072692

[50] Deretic V Saitoh T Akira S Autophagy in infection, inflammation and immunity. Nat Rev Immunol (2013) 13:722–37. 10.1038/nri3532 24064518 PMC5340150

[51] van der Vaart M Korbee CJ Lamers GE Tengeler AC Hosseini R Haks MC The DNA damage-regulated autophagy modulator DRAM1 links mycobacterial recognition via TLR-MYD88 to authophagic defense. Cell Host Microbe (2014) 15:753–67. 10.1016/j.chom.2014.05.005 24922577

[52] Hosseini R Lamers GE Hodzic Z Meijer AH Schaaf MJ Spaink HP. Correlative light and electron microscopy imaging of autophagy in a zebrafish infection model. Autophagy (2014) 10:1844–57. 10.4161/auto.29992 25126731 PMC4198367

[53] Yang CT Cambier CJ Davis JM Hall CJ Crosier PS Ramakrishnan L. Neutrophils exert protection in the early tuberculous granuloma by oxidative killing of mycobacteria phagocytosed from infected macrophages. Cell Host Microbe (2012) 12:301–12. 10.1016/j.chom.2012.07.009 22980327 PMC3638950

[54] Ramakrishnan L. Looking within the zebrafish to understand the tuberculous granuloma. Adv Exp Med Biol (2013) 783:251–66. 10.1007/978-1-4614-6111-1_13 23468113

[55] Oehlers SH Cronan MR Scott NR Thomas MI Okuda KS Walton EM Interception of host angiogenic signalling limits mycobacterial growth. Nature (2015) 517:612–5. 10.1038/nature13967 25470057 PMC4312197

[56] Houben D Demangel C van Ingen J Perez J Baldeon L Abdallah AM ESX-1-mediated translocation to the cytosol controls virulence of mycobacteria. Cell Microbiol (2012) 14:1287–98. 10.1111/j.1462-5822.2012.01799.x 22524898

[57] Cambier CJ Takaki KK Larson RP Hernandez RE Tobin DM Urdahl KB Mycobacteria manipulate macrophage recruitment through coordinated use of membrane lipids. Nature (2014) 505:218–22. 10.1038/nature12799 24336213 PMC3961847

[58] Kaufman CK White RM Zon L Chemical genetic screening in the zebrafish embryo. Nat Protoc (2009) 4:1422–32. 10.1038/nprot.2009.144 19745824 PMC2943144

[59] Takaki K Davis JM Winglee K Ramakrishnan L. Evaluation of the pathogenesis and treatment of *Mycobacterium marinum* infection in zebrafish. Nat Protoc (2013) 8:1114–24. 10.1038/nprot.2013.068 23680983 PMC3919459

[60] Adams KN Takaki K Connolly LE Wiedenhoft H Winglee K Humbert O Drug tolerance in replicating mycobacteria mediated by a macrophage-induced efflux mechanism. Cell (2011) 145:39–53. 10.1016/j.cell.2011.02.022 21376383 PMC3117281

[61] Adams KN Szumowski JD Ramakrishnan L Verapamil, and its metabolite norverapamil, inhibit macrophage-induced, bacterial efflux pump-mediated tolerance to multiple anti-tubercular drugs. J Infect Dis (2014) 210:456–66. 10.1093/infdis/jiu095 24532601 PMC4110457

[62] Torraca V Cui C Boland R Bebelman JP van der Sar AM Smit MJ The CXCR3-CXCL11 signaling axis mediates macrophage recruitment and dissemination of mycobacterial infection. Dis Model Mech (2015) 8:253–69. 10.1242/dmm.017756 25573892 PMC4348563

[63] Tobin DM Roca FJ Oh SF McFarland R Vickery TW Ray JP Host genotype-specific therapies can optimize the inflammatory response to mycobacterial infections. Cell (2012) 148:434–46. 10.1016/j.cell.2011.12.023 22304914 PMC3433720

[64] Roca FJ Ramakrishnan L. TNF dually mediates resistance and susceptibility to mycobacteria via mitochondrial reactive oxygen species. Cell (2013) 153:521–34. 10.1016/j.cell.2013.03.022 23582643 PMC3790588

[65] Kaufmann SH Lange C Rao M Balaji KN Lotze M Schito M Progress in tuberculosis vaccine development and host-directed therapies – a state of the art review. Lancet Respir Med (2014) 2:301–20. 10.1016/S2213-2600(14)70033-5 24717627

[66] Deng W Tang X Hou M Li C Xie J. New insights into the pathogenesis of tuberculosis revealed by *Mycobacterium marinum*: the zebrafish model from the systems biology perspective. Crit Rev Eukaryot Gene Expr (2011) 21:337–45. 10.1615/CritRevEukarGeneExpr.v21.i4.40 22181703

[67] Case ED Samuel JE. Contrasting lifestyles within the host cell. Microbiol Spectr (2016) 4. 10.1128/microbiolspec.VMBF-0014-2015 26999394 PMC4804636

[68] Oksanen KE Halfpenny NJ Sherwood E Harjula SK Hammaren MM Ahava MJ An adult zebrafish model for preclinical tuberculosis vaccine development. Vaccine (2013) 31:5202–9. 10.1016/j.vaccine.2013.08.093 24055305

[69] Ojanen MJ Turpeinen H Cordova ZM Hammaren MM Harjula SK Parikka M The proprotein convertase subtilisin/kexin furinA regulates zebrafish host response against *Mycobacterium marinum*. Infect Immun (2015) 83:1431–42. 10.1128/IAI.03135-14 25624351 PMC4363408

[70] Hammaren MM Oksanen KE Nisula HM Luukinen BV Pesu M Ramet M Adequate Th2-type response associates with restricted bacterial growth in latent mycobacterial infection of zebrafish. PLoS Pathog (2014) 10:e1004190. 10.1371/journal.ppat.1004190 24968056 PMC4072801

[71] van Meijgaarden KE Haks MC Caccamo N Dieli F Ottenhoff TH Joosten SA. Human CD8+ T-cells recognizing peptides from *Mycobacterium tuberculosis* (Mtb) presented by HLA-E have an unorthodox Th2-like, multifunctional, Mtb inhibitory phenotype and represent a novel human T-cell subset. PLoS Pathog (2015) 11:e1004671. 10.1371/journal.ppat.1004671 25803478 PMC4372528

[72] van der Sar AM Abdallah AM Sparrius M Reinders E Vandenbroucke-Grauls CM Bitter W. *Mycobacterium marinum* strains can be divided into two distinct types based on genetic diversity and virulence. Infect Immun (2004) 72:6306–12. 10.1128/IAI.72.11.6306-6312.2004 15501758 PMC523024

[73] Oksanen KE Myllymäki H Ahava MJ Mäkinen L Parikka M Rämet M. DNA vaccination boosts Bacillus Calmette-Guérin protection against mycobacterial infection in zebrafish. Dev Comp Immunol (2016) 54:89–96. 10.1016/j.dci.2015.09.001 26363085

[74] Cui Z Samuel-Shaker D Watral V Kent ML Attenuated *Mycobacterium marinum* protects zebrafish against mycobacteriosis. J Fish Dis (2010) 33:371–5. 10.1111/j.1365-2761.2009.01115.x 19912456 PMC3951474

[75] Stoop EJ Schipper T Rosendahl Huber SK Nezhinsky AE Verbeek FJ Gurcha SS Zebrafish embryo screen for mycobacterial genes involved in the initiation of granuloma formation reveals a newly identified ESX-1 component. Dis Model Mech (2011) 4:526–36. 10.1242/dmm.006676 21372049 PMC3124061

[76] Volkman HE Clay H Beery D Chang JC Sherman DR Ramakrishnan L. Tuberculous granuloma formation is enhanced by a mycobacterium virulence determinant. PLoS Biol (2004) 2:e367. 10.1371/journal.pbio.0020367 15510227 PMC524251

[77] Knudsen NP Norskov-Lauritsen S Dolganov GM Schoolnik GK Lindenstrom T Andersen P Tuberculosis vaccine with high predicted population coverage and compatibility with modern diagnostics. Proc Natl Acad Sci U S A (2014) 111:1096–101. 10.1073/pnas.1314973111 24395772 PMC3903227

[78] Hoang T Aagaard C Dietrich J Cassidy JP Dolganov G Schoolnik GK ESAT-6 (EsxA) and TB10.4 (EsxH) based vaccines for pre- and post-exposure tuberculosis vaccination. PLoS One (2013) 8:e80579. 10.1371/journal.pone.0080579 24349004 PMC3861245

[79] Bottai D Frigui W Clark S Rayner E Zelmer A Andreu N Increased protective efficacy of recombinant BCG strains expressing virulence-neutral proteins of the ESX-1 secretion system. Vaccine (2015) 33:2710–8. 10.1016/j.vaccine.2015.03.083 25869896

[80] Lin PL Dietrich J Tan E Abalos RM Burgos J Bigbee C The multistage vaccine H56 boosts the effects of BCG to protect cynomolgus macaques against active tuberculosis and reactivation of latent *Mycobacterium tuberculosis* infection. J Clin Invest (2012) 122:303–14. 10.1172/JCI46252 22133873 PMC3248283

[81] Dietrich J Andersen C Rappuoli R Doherty TM Jensen CG Andersen P. Mucosal administration of Ag85B-ESAT-6 protects against infection with *Mycobacterium tuberculosis* and boosts prior bacillus Calmette-Guerin immunity. J Immunol (2006) 177:6353–60. 10.4049/jimmunol.177.9.6353 17056566

[82] Yuan W Dong N Zhang L Liu J Lin S Xiang Z Immunogenicity and protective efficacy of a tuberculosis DNA vaccine expressing a fusion protein of Ag85B-Esat6-HspX in mice. Vaccine (2012) 30:2490–7. 10.1016/j.vaccine.2011.06.029 21704108

[83] Capuano SVI Croix DA Pawar S Zinovik A Myers A Lin PL Experimental *Mycobacterium tuberculosis* infection of cynomolgus macaques closely resembles the various manifestations of human *M. tuberculosis* infection. Infect Immun (2003) 71:5831–44. 10.1128/IAI.71.10.5831-5844.2003 14500505 PMC201048

[84] Murray KN Bauer J Tallen A Matthews JL Westerfield M Varga ZM. Characterization and management of asymptomatic mycobacterium infections at the Zebrafish International Resource Center. J Am Assoc Lab Anim Sci (2011) 50:675–9. 22330714 PMC3189671

[85] Pasnik DJ Smith SA. Immunogenic and protective effects of a DNA vaccine for *Mycobacterium marinum* in fish. Vet Immunol Immunopathol (2005) 103:195–206. 10.1016/j.vetimm.2004.08.017 15621306

[86] Kato G Kondo H Aoki T Hirono I. BCG vaccine confers adaptive immunity against *Mycobacterium* sp. infection in fish. Dev Comp Immunol (2010) 34:133–40. 10.1016/j.dci.2009.08.013 19733586

[87] Ernst JD. The immunological life cycle of tuberculosis. Nat Rev Immunol (2012) 12:581–91. 10.1038/nri3259 22790178

[88] Berry MP Graham CM McNab FW Xu Z Bloch SA Oni T An interferon-inducible neutrophil-driven blood transcriptional signature in human tuberculosis. Nature (2010) 466:973–7. 10.1038/nature09247 20725040 PMC3492754

[89] Kaufmann SH Dorhoi A. Inflammation in tuberculosis: interactions, imbalances and interventions. Curr Opin Immunol (2013) 25:441–9. 10.1016/j.coi.2013.05.005 23725875

[90] Fletcher HA. Profiling the host immune response to tuberculosis vaccines. Vaccine (2015) 33:5313–5. 10.1016/j.vaccine.2015.07.090 26241949

[91] Tameris MD Hatherill M Landry BS Scriba TJ Snowden MA Lockhart S Safety and efficacy of MVA85A, a new tuberculosis vaccine, in infants previously vaccinated with BCG: a randomised, placebo-controlled phase 2b trial. Lancet (2013) 381:1021–8. 10.1016/S0140-6736(13)60177-4 23391465 PMC5424647

[92] Kagina BM Abel B Scriba TJ Hughes EJ Keyser A Soares A Specific T cell frequency and cytokine expression profile do not correlate with protection against tuberculosis after Bacillus Calmette-Guérin vaccination of newborns. Am J Respir Crit Care Med (2010) 182:1073 10.1164/rccm.201003-0334OC 20558627 PMC2970848

